# Suspension-Based Intracellular Immunofluorescence Staining Coupled With Genome-Wide CRISPR-Cas9 Screening to Identify Regulators of *O-*GlcNAcylation

**DOI:** 10.21769/BioProtoc.5858

**Published:** 2026-11-05

**Authors:** Peiyu Liu, Qin Lei, Na Zhang, Haibin Yu, Fang Chen, Kai Yuan

**Affiliations:** 1 Institute of Cytology and Genetics, School of Basic Medical Sciences, Hengyang Medical School, University of South China, Hengyang, Hunan, China; 2 Center for Medical Genetics, School of Life Sciences, Central South University, Changsha, China; 3 Hunan Key Laboratory of Molecular Precision Medicine, Department of Oncology, Xiangya Hospital, Central South University, Changsha, China; 4 Furong Laboratory, Changsha, China; 5 National Clinical Research Center for Geriatric Disorders, Xiangya Hospital, Central South University, Changsha, China; 6 The Biobank of Xiangya Hospital, Central South University, Changsha, China

**Keywords:** Protein *O-*GlcNAcylation, GeCKO, Pooled CRISPR screen, Immunofluorescence, Fluorescence-activated cell sorting (FACS), MAGeCK

## Abstract

Protein *O-*GlcNAcylation is a dynamic and reversible post-translational modification that regulates diverse cellular processes, including transcription, signal transduction, metabolism, and cell fate determination. Systematic identification of genes that modulate global *O-*GlcNAc levels remains technically challenging at the genome scale. Here, we describe a pooled CRISPR-Cas9 screening protocol that combines the human genome-scale CRISPR knockout (GeCKO) v2 knockout library with intracellular immunofluorescence staining using the anti-*O-*GlcNAc antibody RL2 and fluorescence-activated cell sorting (FACS). In this workflow, HEK293T cells are transduced with the GeCKO v2 lentiviral library at a low multiplicity of infection to ensure predominantly single-sgRNA integration. Following puromycin selection, cells are fixed, permeabilized, and stained in suspension with RL2. The top 5% of cells with the highest intracellular RL2 fluorescence are collected as the RL2-high population, while a corresponding unsorted/input sample is retained as the reference for downstream sgRNA enrichment analysis. Genomic DNA is recovered from the RL2-high and unsorted/input samples, and integrated sgRNA cassettes are amplified through a two-step PCR and library-preparation workflow for Illumina sequencing. This protocol enables the identification of candidate genes whose knockout is associated with increased intracellular RL2 fluorescence.

Key features

• A genome-scale pooled CRISPR-Cas9 screening workflow using the human GeCKO v2 library in HEK293T cells.

• A suspension-based intracellular RL2 immunofluorescence staining protocol optimized to minimize cell loss during fixation, permeabilization, and washing, thereby preserving sgRNA library representation for downstream sorting.

• Isolation of the top 5% RL2-high population, with a corresponding unsorted/input sample retained as the reference for downstream sgRNA enrichment analysis.

• -Fixed-cell genomic DNA recovery and sgRNA sequencing coupled with model-based analysis of genome-wide CRISPR/Cas9 knockout (MAGeCK) enable robust hit identification.

## Graphical overview


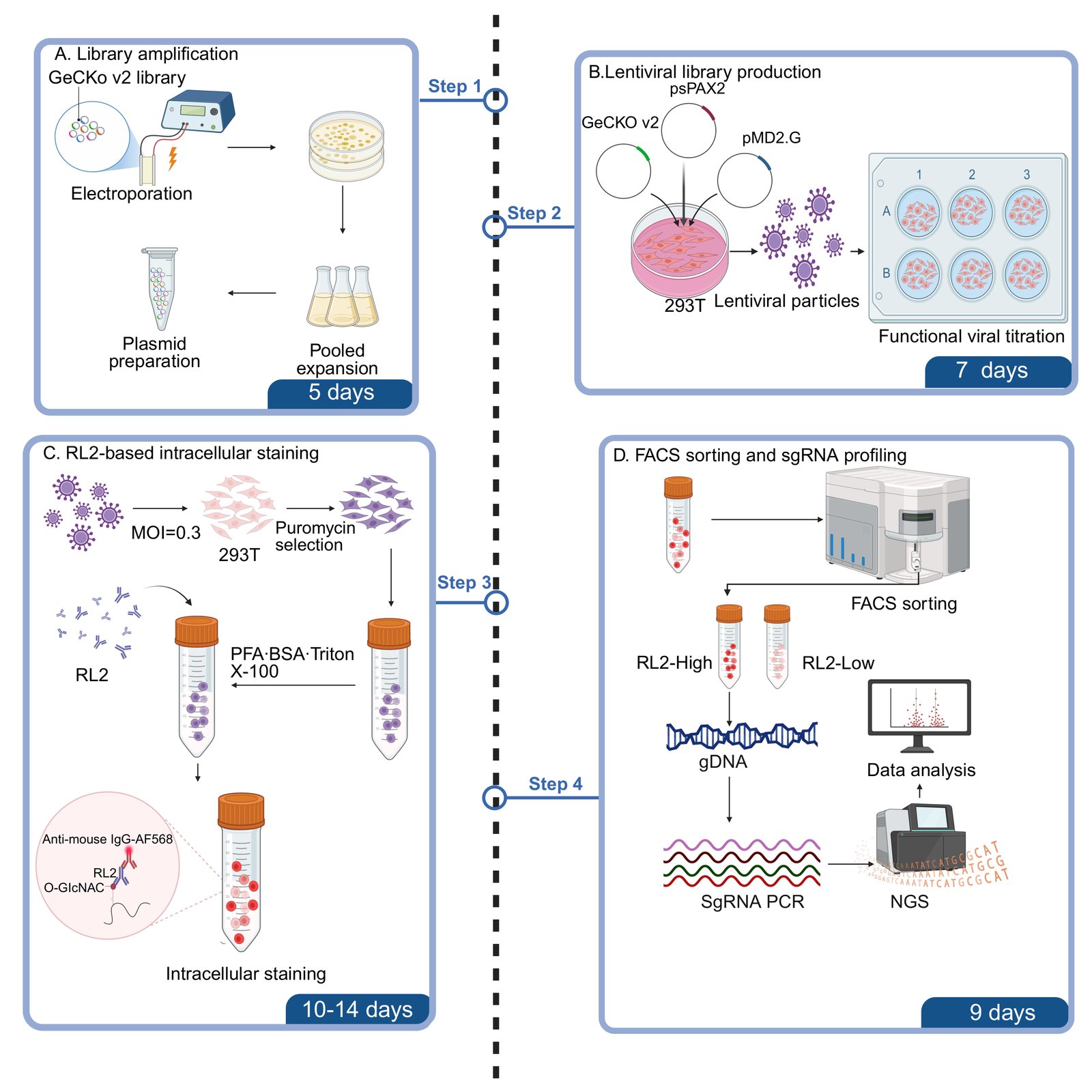


**Workflow of the GeCKO v2–RL2 pooled CRISPR screen.** The human GeCKO v2 library is amplified, packaged into lentivirus, and transduced into HEK293T cells at a low multiplicity of infection (MOI). Following puromycin selection, cells are stained with RL2. The top 5% RL2-high population and a corresponding unsorted/input reference are sequenced and compared using MAGeCK. The RL2-low population shown in the sorting schematic is a fluorescence and sorting control only and is not sequenced.

## Background

Protein *O-*GlcNAcylation is a dynamic and reversible intracellular post-translational modification in which a single *O-*linked β-N-acetylglucosamine moiety is added to serine or threonine residues of nuclear and cytoplasmic proteins by *O-*GlcNAc transferase (OGT) and removed by *O-*GlcNAcase (OGA) [1]. Because UDP-GlcNAc, the donor substrate for OGT, is generated through the hexosamine biosynthetic pathway, cellular *O-*GlcNAcylation is closely coupled to nutrient availability, metabolic state, and stress responses. *O-*GlcNAcylation regulates multiple biological processes, including transcription, chromatin regulation, signal transduction, metabolism, proteostasis, and cell fate decisions. Dysregulation of *O-*GlcNAc homeostasis has been associated with pathological states, including cancer and metabolic disorders [1,2].

Existing approaches for investigating *O-*GlcNAc regulation mainly rely on targeted perturbation and detection strategies, including pharmacological perturbation of *O-*GlcNAc cycling, antibody-based immunodetection, chemoenzymatic labeling, and mass spectrometry–based profiling [2−4]. These approaches are useful for measuring global or protein-specific *O-*GlcNAcylation and for validating candidate regulators. However, they are not designed to identify, in an unbiased genome-scale manner, the broader genetic network that controls intracellular *O-*GlcNAc homeostasis. Therefore, a scalable functional-genomics strategy is needed to systematically discover genes whose loss increases or decreases global *O-*GlcNAcylation.

Pooled CRISPR-Cas9 screening provides a powerful strategy for connecting genetic perturbations with cellular phenotypes. The human GeCKO library established lentiviral genome-scale knockout screening in human cells, and the GeCKO v2 system further improved the vectors and genome-wide libraries for CRISPR screening [5,6]. Subsequent genome-scale CRISPR screening protocols have defined essential experimental principles, including low multiplicity of infection (MOI), antibiotic selection, maintenance of sufficient sgRNA library coverage, and sequencing-based guide recovery [7]. In parallel, phenotype-based and high-content CRISPR screening strategies, together with diverse applications in cancer and immune-cell systems, have expanded pooled screens beyond simple viability readouts to more complex cellular and molecular phenotypes [8−10]. For sortable phenotypes, fluorescence-activated cell sorting (FACS)-based pooled CRISPR screening enables the enrichment of cell populations with high or low levels of a defined fluorescence signal, including antibody-based intracellular molecular readouts [11,12].

Here, we describe a GeCKO v2–RL2 pooled CRISPR screening protocol that converts global *O-*GlcNAcylation into a sortable fluorescence phenotype. HEK293T cells are transduced with the GeCKO v2 lentiviral library at low MOI, selected with puromycin, expanded while maintaining sgRNA representation, fixed and permeabilized in suspension, and stained with the anti-*O-*GlcNAc antibody RL2 followed by an Alexa Fluor 568–conjugated secondary antibody and DAPI. The top 5% of cells with the highest intracellular RL2 fluorescence are collected as the RL2-high population, while a corresponding unsorted/input sample is retained as the reference for downstream sgRNA enrichment analysis. Integrated sgRNA cassettes are recovered from genomic DNA from the RL2-high and unsorted/input samples for next-generation sequencing and MAGeCK-based enrichment analysis [2,13].

By integrating genome-scale perturbation, suspension intracellular immunofluorescence staining, quantitative FACS enrichment, and sgRNA-level computational analysis, this protocol enables unbiased identification of candidate negative regulators whose knockout is associated with increased intracellular RL2 fluorescence. More broadly, the same framework can be adapted to other antibody-defined intracellular phenotypes, including phosphorylation, acetylation, ubiquitination, and chromatin-associated epitopes, providing a general strategy for dissecting intracellular signaling and regulatory networks.

## Materials and reagents

**Biological materials**

1. HEK293T cells (ATCC, catalog number: CRL-3216); cells should be authenticated and routinely tested for mycoplasma contamination

2. Endura electrocompetent *Escherichia coli* cells (Lucigen, catalog number: 60242-2)

**Plasmids and pooled libraries**

1. Human GeCKO v2 CRISPR knockout pooled library in the lentiCRISPR v2 backbone (Addgene pooled library #1000000048); the complete library comprises library A and library B and contains 123,411 sgRNAs targeting 19,050 protein-coding genes, with six sgRNAs per gene and 1,000 non-targeting control sgRNAs

2. pMD2.G lentiviral envelope plasmid (Addgene, plasmid #12259)

3. psPAX2 lentiviral packaging plasmid (Addgene, plasmid #12260)

**Reagents**

1. Dulbecco’s modified Eagle medium (DMEM) (Vivocell, catalog number: 06-1055-57-1 ACS)

2. Fetal bovine serum (FBS) (Meisen, catalog number: CTCC-002-071)

3. 0.25% Trypsin-EDTA (Thermo Fisher Scientific, catalog number: 25200072)

4. Dulbecco’s phosphate-buffered saline (DPBS) without Ca^2+^ and Mg^2+^ (Thermo Fisher Scientific, catalog number: 14190144); critical for minimizing cell aggregation during intracellular staining

5. Accutase cell detachment solution (Innovative Cell Technologies, catalog number: AT-104)

6. Puromycin (Selleck, catalog number: S7417)

7. Hexadimethrine bromide (Polybrene) (Sigma, catalog number: H9268)

8. Opti-MEM I reduced-serum medium (Thermo Fisher Scientific, catalog number: 31985070)

9. Formaldehyde solution, 37% (Sigma, catalog number: 252549)

10. Bovine serum albumin (BSA) (BioFroxx, catalog number: 4240GR005); use ultrapure BSA for reduced flow cytometry background

11. Triton X-100 (Solarbio, catalog number: T8200)

12. Phosphate-buffered saline (PBS) (20× stock) (Servicebio, catalog number: G4202)

13. Anti-*O-*GlcNAc antibody (RL2), mouse monoclonal (Thermo Fisher Scientific, catalog number: MA1-072)

14. Goat anti-mouse immunoglobulin G (IgG) (H+L), Alexa Fluor 568–conjugated (Thermo Fisher Scientific, catalog number: A11004)

15. DAPI (Sigma, catalog number: D9542)

16. Proteinase K (Qiagen, catalog number: 19131)

17. RNase A (Qiagen, catalog number: 19101)

18. Phenol:chloroform:isoamyl alcohol (25:24:1) (Sigma, catalog number: P3803)

19. Chloroform (Sigma, catalog number: C2432)

20. Glycogen (Thermo Fisher Scientific, catalog number: 10814010)

21. KOD DNA polymerase (Merck, catalog number: 71086)

22. dNTP mix (Thermo Fisher Scientific, catalog number: R0192)

23. Polyethylene glycol 8000 (PEG8000) (Solarbio, catalog number: P8260)

24. LB broth (Sigma, catalog number: L3022)

25. Ampicillin sodium salt (Sigma, catalog number: A5354)

26. SOC medium (New England Biolabs, catalog number: B9020S)

27. QIAquick PCR Purification kit (QIAGEN, catalog number: 28104)

28. Qubit^TM^ dsDNA HS Assay kit, 500 assays (Invitrogen, Thermo Fisher Scientific, catalog number: Q32854)

29. NEBNext^®^ Ultra^TM^ II DNA Library Prep Kit for Illumina^®^ (New England Biolabs, catalog number: E7645S)

30. NEBNext^®^ Multiplex Oligos for Illumina^®^ (Index Primers Set 2) (New England Biolabs, catalog number: E7500S)

**Critical:** This product has since been discontinued. According to the manufacturer’s recommendation, the NEBNext^®^ 96 Unique Dual Index Primer Pairs (NEB #E6440S, #E6442S, #E6444S, #E6446S, or #E6448S) can be used as a replacement.

31. Agencourt AMPure XP beads (Beckman Coulter, catalog number: A63881)

32. Penicillin-streptomycin solution (Vivacell, catalog number: C3420-0100)

33. Sodium chloride (NaCl) (Sinopharm, catalog number: 10019318-500g)

**Solutions**

1. Complete growth medium for HEK293T cells (see Recipes)

2. 4% PFA fixative (see Recipes)

3. Blocking/permeabilization buffer (see Recipes)

4. Primary antibody dilution buffer (see Recipes)

5. FACS sorting buffer (see Recipes)

6. Lentivirus storage buffer (see Recipes)

7. 5× PEG8000 virus concentration solution (see Recipes)

**Recipes**

**1. Complete growth medium for HEK293T cells**

Add 50 mL of FBS and 5 mL of 100× penicillin-streptomycin to 445 mL of high-glucose DMEM. Mix thoroughly and store at 4 °C. Use within 1 month.

**2. 4% PFA fixative**

Add 10 mL of 10× PBS to 10.8 mL of 37% formaldehyde. Bring the volume to 100 mL with ddH_2_O. Mix thoroughly. Prepare fresh on the day of use; the working solution is stable for up to 1 week at 4 °C.

**3. Blocking/permeabilization buffer**

Dissolve 2 g of BSA in 5 mL of 20× PBS. Add 100 μL of Triton X-100 and bring the volume to 100 mL with ddH_2_O. Mix thoroughly and filter through a 0.22 μm sterile filter. Store at 4 °C for up to 1 week.

**4. Primary antibody dilution buffer**

Dissolve 0.1 g of BSA in 5 mL of 20× PBS. Add 50 μL of Triton X-100 and bring the volume to 100 mL with ddH_2_O. Mix thoroughly and filter through a 0.22 μm sterile filter. Prepare fresh each time before use.

**5. FACS sorting buffer**

Dissolve 1 g of BSA and 200 μL of 0.5 M EDTA (pH 8.0) in DPBS (Ca^2+^/Mg^2+^-free) and bring the volume to 100 mL with DPBS. Mix thoroughly and filter through a 0.22 μm sterile filter. Keep the solution ice-cold until use.

**6. Lentivirus storage buffer**

Dissolve 10 g of BSA and add 10 mL of FBS to high-glucose DMEM to a final volume of 100 mL. Mix thoroughly and filter through a 0.22 μm sterile filter. Store at -80 °C.

**7. 5× PEG8000 virus concentration solution**

Dissolve 40 g of PEG8000 and 8.77 g of NaCl in ddH_2_O to a final volume of 100 mL. Autoclave to sterilize. Warm gently to dissolve before use and store at 4 °C.

## Equipment

1. Flow cytometer (BD Biosciences, model: FACSAria III)

2. CO_2_ cell culture incubator (Thermo Fisher Scientific, model: 3111)

3. Confocal laser scanning microscope (Zeiss, model: LSM880)

4. Fluorescence stereomicroscope (Leica, model: M205 FCA)

5. Class II A2 biosafety cabinet (Thermo Fisher Scientific)

6. High-speed refrigerated centrifuge (Thermo Fisher Scientific, model: 75004530)

7. Microcentrifuge (Eppendorf, model: 5424R)

8. Electroporator (Bio-Rad, model: Gene Pulser Xcell)

9. NanoDrop spectrophotometer (Thermo Fisher Scientific, model: 2000C)

10. Electroporation cuvettes, 0.1 cm (Bio-Rad, model: 1652089)

## Software and datasets

1. MAGeCK, version 0.5.9

2. R, version 4.0.3

3. FlowJo or equivalent flow cytometry analysis software, version 10.7.1

4. GraphPad Prism, version 8.0

5. BioRender

## Procedure

**A. GeCKO v2 library amplification and plasmid preparation (timing: 5 days)**

**Critical:** Maintain adequate sgRNA representation during bacterial amplification. For the combined GeCKO v2 A and B libraries containing 123,411 sgRNAs, 100× and 300× coverage require approximately 1.23 × 10^7^ and 3.70 × 10^7^ independent transformants, respectively. For more stringent 300× representation, at least 3.7 × 10^7^ independent transformants are required. This section follows the general principles of the GeCKO v2 library amplification protocol, with emphasis on preserving sgRNA representation during bacterial transformation, pooled expansion, and plasmid preparation.

**A1. Electroporation and bacterial recovery**

**Critical:** High-efficiency electrocompetent cells are required for amplification of the genome-wide GeCKO v2 pooled library. Chemically competent cells are not recommended because their lower transformation efficiency may generate an insufficient number of independent transformants, resulting in the loss of low-abundance sgRNAs and reduced library representation. Endura ElectroCompetent *E. coli* cells, as used in this protocol, are recommended [6,7].

1. Thaw Endura electrocompetent cells on ice for approximately 10 min until just melted. Meanwhile, equilibrate SOC medium to room temperature, prewarm 15 cm LB agar plates containing 100 μg/mL ampicillin at 37 °C for 30 min, and pre-chill 0.1 cm electroporation cuvettes and 1.5 mL microcentrifuge tubes on ice. Fresh agar plates are strongly recommended, as aged plates can significantly reduce transformation efficiency.

2. For each electroporation, aliquot 25 μL of Endura cells into a pre-chilled microcentrifuge tube, add 2 μL of GeCKO v2 plasmid DNA (50 ng/μL), and mix gently by pipetting 2–3 times while avoiding bubbles. Transfer the mixture into a pre-chilled 0.1 cm electroporation cuvette, tap gently to settle the suspension, and electroporate at 1,600 V, 200 Ω, and 25 μF.

3. Confirm that the time constant is approximately 4.0 ms.

**Critical:** If the time constant is <3.5 ms, discard the reaction and repeat with fresh competent cells.

4. Immediately add 1 mL of SOC medium to the cuvette, pipette at least three times to fully recover the cells, transfer the suspension into a 14 mL culture tube, rinse the cuvette with an additional 1 mL of SOC, and combine the recovered cells.

5. Repeat the electroporation four additional times for a total of five parallel reactions, then pool all recovered cells into one 50 mL conical tube (Culture A) and incubate at 37 °C, 250 rpm, for 1 h.

**A2. Transformation efficiency and bacterial expansion**

1. Thoroughly mix the recovered bacterial suspension. Transfer 2 μL of the recovered electroporation mixture into 998 μL of SOC medium as the initial dilution. From this suspension, prepare 100-fold, 1,000-fold, and 10,000-fold dilutions in SOC medium. Plate 100 μL of each dilution onto prewarmed LB agar plates containing 100 μg/mL ampicillin.

**Critical:** Lower incubation temperature reduces satellite colony formation and improves colony counting accuracy.

2. Incubate the plates at 32 °C for 16 h.

3. Select a dilution plate containing a countable number of well-separated colonies and use it to estimate the total number of independent transformants. Record the complete dilution procedure, the plated volume, and the total volume of the recovered bacterial suspension.

4. Count colonies from plates containing 30–300 colonies and calculate the total number of independent transformants.

**Critical:** Transformation efficiency should be ≥1 × 10^9^ cfu/μg DNA, corresponding to at least 100× library coverage.

5. Transfer the remaining Culture A into 500 mL of LB broth supplemented with 100 μg/mL ampicillin and incubate at 37 °C, 250 rpm, for 16 h.

**Pause point:** The bacterial culture may be stored at 4 °C for up to 1 week before plasmid extraction.

**A3. Maxi plasmid extraction**

1. Harvest the bacterial culture by centrifugation at 6,000× *g* for 15 min at 4 °C, discard the supernatant completely, and resuspend the pellet in 125 mL of Buffer P1 supplemented with RNase A.

2. Add 125 mL of Buffer P2, gently invert 4–6 times, and incubate at room temperature for 5 min until the lysate becomes clear.

**Critical:** Do not exceed 5 min, as over-lysis may compromise plasmid integrity and bias sgRNA representation.

3. Add 125 mL of pre-chilled Buffer P3, mix immediately by vigorous inversion until fully neutralized, and incubate on ice for 30 min.

4. Clarify the lysate by centrifugation at 20,000× *g* for 30 min at 4 °C, transfer the supernatant to a fresh bottle, and centrifuge again at 20,000× *g* for 15 min at 4 °C.

5. Equilibrate a QIAGEN-tip 500 column with 10 mL of Buffer QBT, load the clarified lysate by gravity flow, wash the column twice with 30 mL of Buffer QC, and elute plasmid DNA with 15 mL of Buffer QN.

**A4. DNA precipitation and quality control**

1. Add 10.5 mL of isopropanol to the eluate, mix thoroughly, and centrifuge at 15,000× *g* for 30 min at 4 °C.

2. Wash the DNA pellet once with 5 mL of 70% ethanol, air-dry for 10 min, and dissolve in 1 mL of TE buffer.

**Expected result:** The final plasmid yield is typically 2–5 mg of total GeCKO library DNA.

3. Measure DNA concentration using NanoDrop spectrophotometry.

**Critical:** The A260/A280 ratio should be 1.8–2.0.

4. Verify plasmid integrity by agarose gel electrophoresis before lentiviral packaging and store purified DNA at -20 °C.

**B. Lentiviral production and transduction optimization (timing: 7 days)**

**Critical:** Perform all lentiviral procedures in a BSL-2 laboratory using appropriate personal protective equipment.

**B1. Lentiviral packaging and concentration**

1. Passage HEK293T cells from a healthy stock culture and seed 4 × 10^6^ cells per 10 cm dish in 10 mL of complete DMEM medium. Incubate overnight at 37 °C with 5% CO_2_.

**Critical:** Cells should reach approximately 70% confluency on the day of transfection.

2. For each 10-cm dish, prepare the plasmid mixture according to a pMD2.G: psPAX2: GeCKO library plasmid mass ratio of 1:2:3, using 2 μg of pMD2.G, 4 μg of psPAX2, and 6 μg of GeCKO library plasmid, for a total of 12 μg of plasmid DNA.

3. Dilute the 12-μg plasmid mixture in 1,200 μL of Opti-MEM. In a separate tube, dilute 12 μL of Lipofectamine 2000 in 1,200 μL of Opti-MEM. Incubate both tubes at room temperature for 5 min.

4. Combine the diluted plasmid mixture with the diluted Lipofectamine 2000 solution, mix gently, and incubate at room temperature for 20 min.

5. Add the transfection mixture dropwise to the HEK293T cells. Gently rock the dish to distribute the mixture evenly and return the cells to the 37 °C, 5% CO_2_ incubator.

**Critical:** Add the transfection mixture slowly along the wall of the dish to avoid detaching HEK293T cells.

6. Replace the medium with 10 mL of fresh complete DMEM medium 12 h after transfection.

7. Collect viral supernatant at 48 and 72 h after transfection. Centrifuge the collected supernatant at 180× *g* for 5 min to remove cell debris and transfer the clarified viral supernatant to fresh tubes.

8. Add 5× PEG8000 virus concentration solution to the clarified viral supernatant at a 5:1 ratio of viral supernatant to 5× PEG8000 solution. Mix thoroughly and incubate at 4 °C overnight with rotation.

9. Centrifuge at 12,000× *g* for 1 h at 4 °C to pellet the virus. Carefully remove the supernatant.

10. Resuspend the viral pellet in an appropriate volume of lentivirus storage buffer or sterile PBS, aliquot into microcentrifuge tubes, and store at -80 °C.

**B2. Puromycin kill curve determination**

1. Seed 3 × 10^5^ HEK293T cells per well in a 6-well plate and incubate overnight.

2. Replace the medium with fresh complete medium containing puromycin at final concentrations of 0, 0.5, 1.0, 1.5, 2.0, and 3.0 μg/mL.

3. Replace with fresh puromycin-containing medium every 48 h and monitor cell viability and morphology daily.

**Critical:** The optimal puromycin concentration should be determined for each cell line before large-scale pooled screening.

**Expected result:** For HEK293T cells, 1 μg/mL puromycin for three days typically achieves complete killing of non-transduced cells.

**Critical:** The optimal puromycin concentration should be determined for each cell line before large-scale pooled screening. The puromycin concentration described here is optimized for HEK293T cells under the specific conditions used in this protocol and should be considered as a reference condition. Since puromycin activity may vary between different lots, a new puromycin kill curve should be performed when using a new batch of puromycin or when applying this protocol to a different cell line or experimental condition.

**B3. Lentiviral titration and MOI determination**

1. Seed 4 × 10^6^ HEK293T cells per 10 cm dish, preparing duplicate dishes for each viral dose.

2. When cells reach approximately 50% confluency, add concentrated lentivirus at the following volumes: 0, 1.25, 2.5, 5, 10, 20, 30, and 40 μL per dish. Add polybrene to a final concentration of 8 μg/mL and incubate for 24 h.

3. Dissociate the cells using trypsin and split each condition into two replicate dishes: one maintained without puromycin and one cultured in medium containing 1 μg/mL puromycin.

4. Continue selection for three days, then count surviving cells from both conditions.

5. Calculate the survival rate as:

Survival rate = Cells with puromycin/Cells without puromycin

6. Calculate the MOI using the following equation:

MOI = -ln (1-survival rate)

**Critical:** Maintain MOI = 0.3–0.5 to ensure predominantly single-sgRNA integration per cell.

**Expected result:** In this workflow, 20 μL of concentrated lentivirus per 10 cm dish typically yields MOI ≈ 0.35, which is suitable for pooled CRISPR screening.

**C. Large-scale pooled GeCKO screening and RL2-based intracellular staining (timing: 10–14 days)**

**Critical:** For the combined human GeCKO v2 A and B libraries (122,411 targeting sgRNAs), a minimum of 3.67 × 10^7^ successfully transduced cells is required to achieve 300× representation of the sgRNA library after puromycin selection. In this experiment, the transduction efficiency was approximately 29%; therefore, approximately 1.27 × 10^8^ cells were used for lentiviral transduction. This 300× coverage applies to the puromycin-selected population before FACS enrichment. The number of cells loaded onto the sorter should be scaled up further if a higher yield of RL2-high cells is required for downstream genomic DNA extraction.

**C1. Large-scale pooled GeCKO screening**

1. Dissociate HEK293T cells from four healthy 10 cm dishes at approximately 80% confluency using trypsin, neutralize with complete medium, and determine viable cell number by trypan blue exclusion.

2. Resuspend the cells in complete medium at the appropriate density and seed 4 × 10^6^ cells in 10 mL per 10 cm dish into 16 dishes total. Incubate overnight at 37 °C with 5% CO_2_.

**Critical:** A total of 6.4 × 10^7^ cells were seeded into 16 dishes on day 1. After overnight expansion, this provided approximately 1.27 × 10^8^ cells for lentiviral transduction. Based on the experimentally determined transduction efficiency of 29%, approximately 3.67 × 10^7^ puromycin-resistant cells were expected to be obtained, corresponding to approximately 300× representation of the combined GeCKO v2 A and B libraries.

3. The next day, confirm that the cells are healthy and suitable for lentiviral transduction. Estimate the total cell number using a representative dish and confirm that the expanded cell population is sufficient for the calculated input of approximately 1.27 × 10^8^ cells. Thaw the concentrated GeCKO lentivirus on ice and mix gently by flicking.

4. Add 20 μL of concentrated GeCKO lentivirus and polybrene to a final concentration of 8 μg/mL to each dish, gently swirl to distribute evenly, and incubate for 24 h at 37 °C with 5% CO_2_.

**Critical:** Avoid disturbing the dishes during the first 8–12 h post-transduction, as movement may reduce infection uniformity.

5. At 24 h post-transduction, carefully aspirate the virus-containing medium and replace with 10 mL of fresh complete medium per dish.

**Expected result:** Cells should remain adherent, morphologically healthy, and typically reach approximately 90% confluency.

6. Begin selection by adding puromycin to a final concentration of 1 μg/mL. Maintain one non-transduced control dish without puromycin to monitor baseline cell growth.

7. Continue selection in puromycin-containing medium, replacing with fresh medium every 48 h.

**Expected result:** By days 6–7, puromycin-resistant cells should begin expanding rapidly, and by day 8, most dishes should recover to 70%–80% confluency.

8. If any dish becomes overconfluent, split the cells into additional puromycin-containing dishes while retaining the entire selected population and recording the viable cell number after each passage.

**Critical:** Do not allow cultures to become overconfluent, as this may introduce growth bias and distort sgRNA enrichment.

9. Once the selected population reaches approximately 80% confluency, proceed immediately to the suspension-based RL2 intracellular staining workflow.

**Critical:** Use freshly proliferating cells for RL2 staining, as overgrown or stressed cultures may artificially alter global *O-*GlcNAc levels.

10. Count and record the number of viable puromycin-resistant cells before subsequent expansion and RL2 staining.

**C2. Suspension intracellular RL2 immunofluorescence staining**

1. Aspirate the culture medium from all selected dishes, wash each dish once with 10 mL of DPBS, then dissociate cells using 1 mL of trypsin per dish for 3–5 min at 37 °C.

2. Neutralize trypsin with 5 mL of complete medium per dish, gently pipette to obtain a single-cell suspension, and pool all cells into 50 mL conical tubes.

3. Centrifuge at 500× *g* for 5 min at 4 °C, discard the supernatant, resuspend the pellet in 10 mL of DPBS, and determine viable cell number before fixation.

4. Aliquot cells into parallel 50 mL tubes for staining. Each tube may contain up to 3 × 10^7^ cells to avoid excessive cell density.

5. Following centrifugation, carefully remove the supernatant and resuspend the cell pellet in freshly prepared 4% PFA. Incubate for 15 min at room temperature with gentle orbital shaking.

6. Centrifuge at 800× *g* for 10 min at 4 °C, remove the fixative completely, and wash the cells twice with DPBS.

**Critical:** Do not extend fixation beyond 20 min, as excessive crosslinking can reduce RL2 epitope accessibility and compress fluorescence dynamic range. NH_4_Cl-mediated quenching is optional in this protocol.

7. Resuspend the pellet in 10 mL of blocking buffer and incubate for 1 h at room temperature on an orbital shaker.

8. Prepare the primary antibody solution in primary antibody dilution buffer using anti-*O-*GlcNAc RL2 at a 1:300 dilution. Optional co-staining antibodies may be included as needed.

9. Pass the antibody solution through a 0.22 μm filter before use.

**Critical:** Filtering the antibody solution markedly reduces clumping during downstream FACS sorting.

10. Resuspend each pellet in 500 μL of primary antibody solution per 3 × 10^6^ cells and incubate at 4 °C overnight (16–18 h) on a rotating shaker.

**Critical:** Overnight incubation is important for achieving sufficient intracellular RL2 signal resolution for subsequent FACS sorting.

11. Wash the cells three times using 1% BSA + 0.1% Triton X-100 in DPBS.

12. Incubate the cells in secondary antibody solution (goat anti-mouse IgG Alexa Fluor 568, 1:300 dilution) supplemented with DAPI (1 μg/mL final) for 45 min at room temperature, protected from light.

**Critical:** DAPI staining is optional. It can be used as a nuclear counterstain to facilitate microscopic visualization and assessment of staining quality, but it is not required for FACS sorting or downstream sgRNA screening analysis.

13. Wash the cells three times with DPBS containing 1% BSA, then resuspend in 0.5–1 mL of FACS sorting buffer.

14. Immediately pass the stained cells through a 40 μm cell strainer before sorting.

**Critical:** This step is essential to remove aggregates and prevent sorter clogging.

15. Before sorting, remove a 20 μL aliquot of the stained cell suspension, prepare a wet mount on a glass slide, and examine the staining quality using a fluorescence microscope.

**Expected result:** More than 90% of cells should display clear intracellular, predominantly nuclear RL2 *O-*GlcNAc fluorescence, as shown in Figure 1.

**Critical:** Samples showing weak signal, high background, or severe aggregation should be optimized before sorting.

16. Keep the samples at 4 °C and protected from light until sorting.

**Pause point:** Cells should be sorted within 2 h after staining completion to preserve fluorescence resolution.

**Figure 1.**
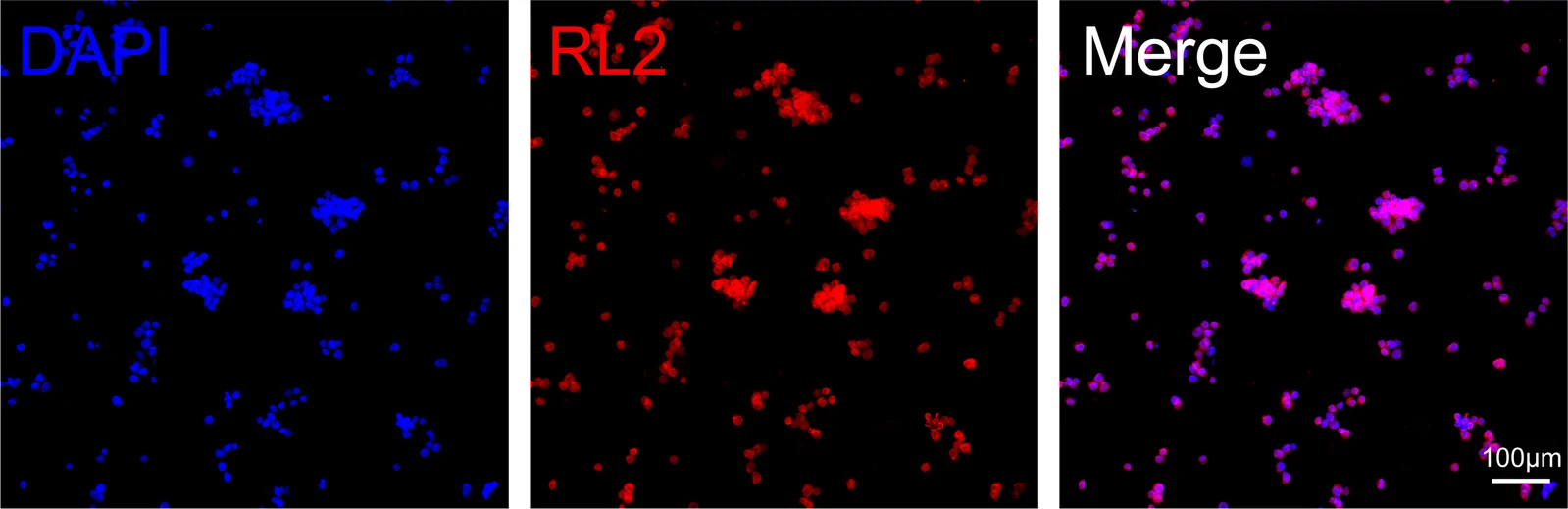
Intracellular RL2 staining of *O-*GlcNAcylated proteins. Representative images of cells stained with RL2 antibody (red) and DAPI (blue). Scale bar, 100 μm.

**D. FACS-based sorting, sgRNA recovery, and sequencing analysis (timing: 9 days)**

**Critical:** Record the number of RL2-high cells recovered and calculate the achieved post-sort sgRNA representation.

**D1. FACS sorting of the RL2-high population**

*Note: Configure the flow cytometer with a 561 nm laser to excite the Alexa Fluor 568–conjugated secondary antibody and a 405 nm laser to excite DAPI. Select the corresponding detector and filter settings according to the instrument configuration.*

1. Adjust forward scatter (FSC) and side scatter (SSC) voltages to clearly separate intact cells from debris and establish a stringent singlet gating strategy using FSC-A vs. FSC-H, with optional SSC-A vs. SSC-W gating to remove doublets and aggregates.

**Critical:** Stringent singlet gating is essential to prevent false enrichment caused by cell clumps.

2. Run the unstained control sample first to establish baseline fluorescence, then analyze the stained screening population and define sorting gates based on the RL2 fluorescence histogram.

3. Before sorting, retain an aliquot of the stained cell suspension as the unsorted/input sample. Sort cells within the top 5% and bottom 5% of the RL2 fluorescence distribution as the RL2-high and RL2-low populations, respectively. The RL2-low population is collected only as a fluorescence control during FACS to confirm the separation of the RL2-high population and is not used for sequencing. Of the two sorted populations, only the RL2-high population is subjected to sgRNA sequencing and downstream analysis, with the unsorted/input sample serving as the reference.

4. Collect the RL2-high population and RL2-low control into separately labeled 1.5 mL low-retention tubes containing 500 μL of complete medium supplemented with 20% FBS. Keep the unsorted/input sample in a separately labeled tube.

5. Collect RL2-high cells for genomic DNA extraction. Based on our experimental records, approximately 2 × 10^6^ cells yielded sufficient genomic DNA for PCR amplification and library preparation. The actual number of recovered cells should be recorded; if recovery is insufficient, increase the number of cells loaded onto the sorter in subsequent experiments.

6. Immediately examine small aliquots of the sorted RL2-high and RL2-low populations under a fluorescence microscope to confirm the sorting result. The RL2-high cells should exhibit clear RL2 fluorescence, whereas the RL2-low cells should show minimal signal. Representative FACS sorting and fluorescence results are shown in Figure 2A, B.

7. Proceed directly to genomic DNA extraction from the RL2-high and unsorted/input samples.

**D2. Genomic DNA extraction from RL2-high and unsorted/input samples**

1. Centrifuge the RL2-high and unsorted/input samples separately at 500× *g* for 5 min at 4 °C, remove the supernatant completely, and resuspend the pellet in LB3 lysis buffer at a density of ≤3 × 10^7^ cells/mL.

2. Add RNase A to a 0.2 mg/mL final concentration and incubate at 65 °C for 4 h or overnight.

**Critical:** Complete lysis is essential for preserving equal sgRNA representation across the RL2-high and unsorted/input samples.

3. Add proteinase K to a 0.2 mg/mL final concentration and continue incubation at 65 °C overnight in a hybridization oven or thermomixer.

**Critical:** Because the cells are PFA-fixed, digestion should be extended to at least 12 h to ensure efficient reversal of crosslinks.

4. Extract the lysate twice using an equal volume of phenol:chloroform:isoamyl alcohol (25:24:1), followed by one chloroform cleanup step.

5. Transfer the aqueous phase to a fresh tube, add 1/10 volume 3 M sodium acetate (pH = 5.2), 1.5 μL of glycogen, and 2 volumes of absolute ethanol; then, precipitate at -80 °C for 15 min.

6. Centrifuge at 6,000× *g* for 10 min at 4 °C.

**Critical:** The DNA pellet is often invisible. Always orient the tube hinge outward and avoid aspirating the pellet.

7. Wash the pellet twice with 75% ethanol, air-dry for 10 min, and dissolve in 50 μL of TE buffer or nuclease-free water.

**Pause point:** Purified genomic DNA can be stored at -20 °C for several months before sgRNA amplification.

**D3. sgRNA amplicon PCR and sequencing library construction**

1. Amplify the integrated GeCKO v2 sgRNA cassette from genomic DNA from the RL2-high and corresponding unsorted/input samples using KOD DNA polymerase and the PCR1 forward and reverse primers. Distribute the genomic DNA from each sample across multiple parallel 50 μL PCR1 reactions, with 2–3 μg of genomic DNA per reaction.

2. Perform PCR1 using the following cycling program: 94 °C for 2 min; a maximum of 30 cycles of 98 °C for 10 s, 55 °C for 30 s, and 68 °C for 10 s; followed by 68 °C for 5 min and a hold at 4 °C.

3. Pool all PCR1 products from the same sample and load 5 μL on an agarose gel to confirm specific amplification. Purify the remaining PCR products using the QIAquick PCR Purification kit. Elute the purified DNA in 60 μL of nuclease-free water and quantify it using the Qubit dsDNA HS Assay.

4. Use 1 μg of purified PCR1 product for library preparation with the NEBNext Ultra II DNA Library Prep Kit for Illumina. Add 3 μL of NEBNext Ultra II End Prep Enzyme Mix and 7 μL of End Prep Reaction Buffer to 50 μL of purified DNA. Incubate at 20 °C for 30 min and 65 °C for 30 min.

5. Add 30 μL of NEBNext Ultra II Ligation Master Mix, 1 μL of Ligation Enhancer, and 2.5 μL of NEBNext Adaptor for Illumina. Incubate at 20 °C for 15 min. Add 3 μL of USER Enzyme and incubate at 37 °C for 15 min.

**Expected result:** Typical concentration is 100–200 ng/μL, corresponding to approximately 5–10 μg of total DNA per sample.

6. Purify the adaptor-ligated DNA using 87 μL of AMPure XP beads. Incubate for 5 min at room temperature, place the tube on a magnetic rack, wash the beads twice with freshly prepared 80% ethanol, and elute the DNA in 17 μL of nuclease-free water. Transfer 15 μL eluate to a fresh PCR tube.

7. Assign a unique index to each RL2-high sample and its corresponding unsorted/input sample. Set up PCR2 in a 50 μL reaction containing 15 μL of adaptor-ligated DNA, 25 μL of NEBNext Ultra II Q5 Master Mix, and 10 μL of indexing primers. In the original experiment, E7500S was used with 5 μL of Index Primer and 5 μL of Universal PCR Primer. If using the currently available E6440S series (96 Unique Dual Index Primer Pairs), add 10 μL of the premixed dual-index primer pair. Follow the manufacturer’s instructions for the specific kit used.

8. Perform PCR2 using the following program: 98 °C for 30 s; 2 cycles of 98 °C for 10 s and 65 °C for 75 s; 65 °C for 5 min; hold at 4 °C.

9. Purify the indexed PCR2 product using 45 μL of AMPure XP beads. Wash the beads twice with 80% ethanol and elute the final library in 33 μL of nuclease-free water. Quantify the final library using the Qubit dsDNA HS Assay.

*Note: In the original experiment, libraries were sequenced using paired-end 75-bp sequencing (PE75), with approximately 10 million reads per sample.*

**Figure 2.**
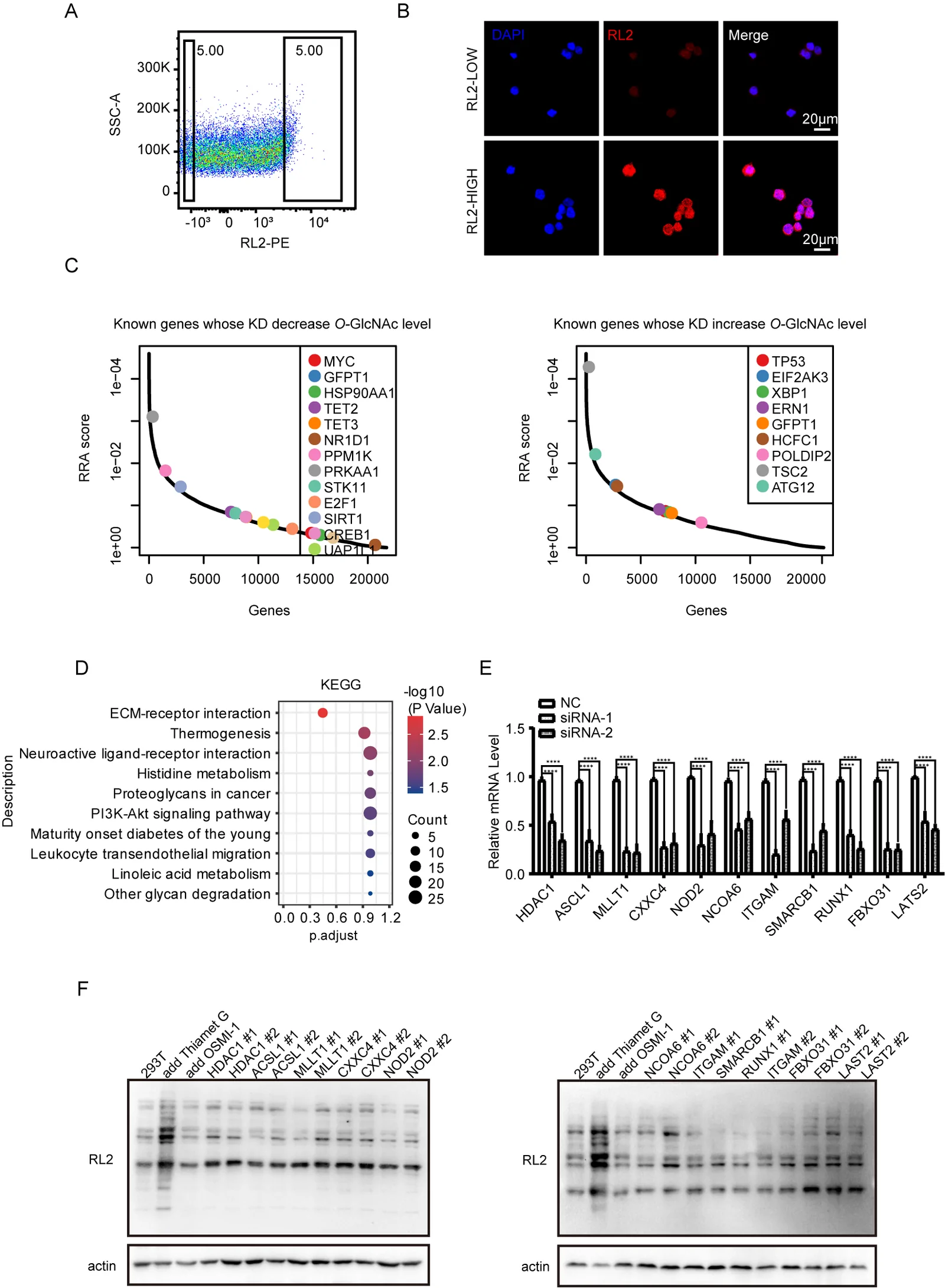
FACS-based enrichment, sgRNA analysis, and validation of candidates identified from the RL2-based pooled CRISPR screen. (A) Flow cytometry analysis of RL2 fluorescence intensity in cells transduced with the GeCKO v2 library and the gating strategy used to isolate the top 5% RL2-high population. The RL2-low population was collected as a fluorescence control. (B) Representative fluorescence images of sorted RL2-high and RL2-low populations stained with RL2 antibody (red) and DAPI (blue), demonstrating the difference in intracellular *O-*GlcNAc signal intensity. Scale bar, 20 μm. (C) Ranking of known *O-*GlcNAcylation regulators by MAGeCK robust rank aggregation (RRA) enrichment scores from the RL2-high vs. input comparison. Genes known to decrease *O-*GlcNAcylation upon knockdown (left) and genes known to increase *O-*GlcNAcylation upon knockdown (right) are highlighted. Genes with lower RRA scores are more strongly enriched in the RL2-high population. (D) Kyoto Encyclopedia of Genes and Genomes (KEGG) pathway enrichment analysis of genes identified as significantly enriched in the RL2-high population. Top enriched pathways are shown. The color scale and dot size indicate the significance and gene count, respectively. (E) Validation of candidate hits by siRNA-mediated knockdown. Cells were transfected with control siRNA (NC) or siRNA targeting the indicated gene(s). Data are presented as mean ± standard error of the mean (SEM). (F) Western blot analysis confirming the changes in global *O-*GlcNAcylation upon knockdown of candidate genes. RL2 antibody was used to detect *O-*GlcNAcylated proteins; actin served as a loading control.

## Data analysis

The RL2-based genome-wide CRISPR screen was performed in two independent runs. In each screen, the top 5% RL2-high population was collected, and the corresponding unsorted/input sample was retained as the reference for sgRNA enrichment analysis. MAGeCKFlute was used for downstream analysis, including quality control, gene-level ranking, and KEGG pathway enrichment analysis. The overall MAGeCK-based data analysis workflow is summarized in Figure 3.

**Figure 3.**
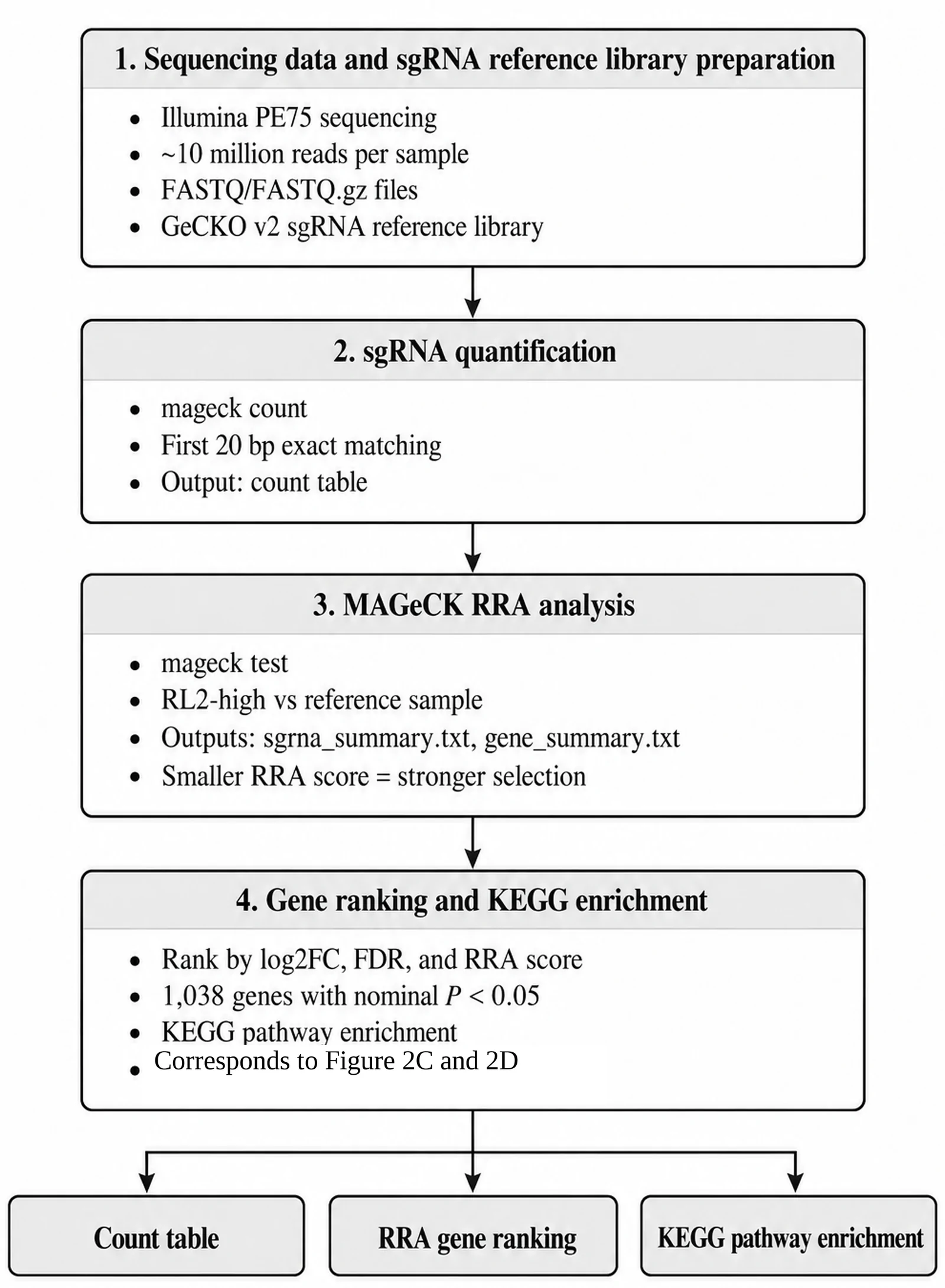
Schematic workflow of the MAGeCK-based data analysis. The flowchart illustrates the major steps of the bioinformatics analysis following the RL2-based FACS screening. (1) Sequencing data and sgRNA reference library preparation: libraries were sequenced using Illumina PE75 with approximately 10 million reads per sample. FASTQ files were used as input along with the GeCKO v2 sgRNA reference library. (2) sgRNA quantification: read counts were generated using *mageck count* by exact matching to the first 20 bp of each sgRNA sequence. The output is a read count table. (3) MAGeCK RRA analysis: the *mageck test* was used to compare the RL2-high population against the unsorted reference sample. The output includes *sgRNA_summary.txt* and *gene_summary.txt*; a smaller RRA score indicates stronger enrichment. (4) Gene ranking and KEGG enrichment: significantly enriched genes (nominal P < 0.05) were ranked by RRA score, log2 fold change, and FDR, followed by KEGG pathway enrichment analysis. The corresponding results are shown in Figure 2C, D.

**1. Sequencing data and sgRNA reference library preparation**

Before starting the analysis, prepare the corresponding sgRNA reference-library file. The human genome-scale CRISPR knockout library used in this protocol was the GeCKO v2 library (Addgene #1000000048) in the lentiCRISPR v2 vector (Addgene #52961), which contains 123,411 sgRNAs in total, including approximately 122,411 sgRNAs targeting 19,050 protein-coding genes and 1,000 non-targeting control sgRNAs [2]. A MAGeCK-compatible human GeCKO v2 reference-library file can be obtained from the MAGeCK SourceForge repository. The sgRNA library file can be provided in .txt or .csv format, with the first three columns corresponding to the sgRNA identifier, sgRNA sequence, and target gene, respectively.

**2. sgRNA quantification and generation of the read-count table**

Use the *mageck count* subcommand to map sequencing reads to the sgRNA reference library and count the sequencing reads corresponding to each sgRNA. In the original RL2 screen, sequencing reads were counted for each guide by taking the first 20 bp from each read and mapping them to the corresponding sgRNA sequence in the GeCKO v2 library [2].

Place the FASTQ/FASTQ.gz files and the sgRNA library file in an accessible working directory, and run *mageck count* using the following general command:

*mageck count \*

*-l <sgRNA_library_file> \*

*-n <output_prefix> \*

*--sample-label < RL2pos >, <input> \*

*--fastq< RL2pos1_R1.fastq.gz>,< input1_R2.fastq.gz> < RL2pos2_R1.fastq.gz>,< input2_R2.fastq.gz>\*

*--fastq-2< RL2pos-1_R2.fastq.gz>,< RL2pos-2-R2.fastq.gz> < input-1_R2.fastq.gz>,< input-2_R2.fastq.gz>*

The parameters are defined as follows:

*-l*: the sgRNA reference-library file containing the sgRNA ID, sequence, and target gene.

*-n*: the prefix assigned to the output files.

*--sample-label*: the sample labels, listed in the same order as the corresponding FASTQ files.

*--fastq*: the paired-end FASTQ files (Read 1 and Read 2) used for sgRNA counting (supports both .*fastq* and .*fastq.gz* formats).

Additional parameters can be viewed by running *mageck count -h*.

**Critical:** For paired-end sequencing data, identify which read contains the sgRNA sequence based on the library design and provide the corresponding FASTQ file(s) for *mageck count*. In the GeCKO v2 library with the lentiCRISPR v2 backbone, the sgRNA sequence is typically contained in Read 1 (R1). However, users should confirm this based on their specific sequencing layout and should not assume the sgRNA is in a particular read without verification.

If *mageck count* runs successfully, the primary read-count output is:

*<output_prefix>.count.txt*

Each row corresponds to one sgRNA, and the sample columns contain the number of reads assigned to that sgRNA. This count table is used directly as the input for subsequent MAGeCK RRA analysis.

MAGeCK also generates *<output_prefix>.countsummary.txt*, which summarizes basic counting and mapping information for the input samples.

**3. MAGeCK Robust Rank Aggregation analysis**

Use the sgRNA read-count table generated in the previous step as the input for MAGeCK RRA analysis. The *mageck test* subcommand performs sgRNA- and gene-level ranking based on the supplied read-count table.

Run *mageck test* using the following general command:

*mageck test \*

*-k <count_table.txt> \*

*-t <treatment_sample1>,<treatment_sample2> \*

*-c <control_sample1>,<control_sample2> \*

*-n <output_prefix>*

The parameters are defined as follows:

*-k*: the sgRNA read-count table generated in the previous step.

*-t*: the phenotype-enriched sample(s) (RL2-high population).

*-c*: the corresponding control/reference sample(s) (input).

*-n*: the prefix assigned to the output files.

Sample labels specified with *-t* and *-c* must match the labels in the header of the input count table. When biological replicates are available, sample labels can be supplied as comma-separated values. By default, MAGeCK uses median normalization and Benjamini–Hochberg FDR correction for multiple testing; additional parameters (*--norm-method, --adjust-method*) can be viewed with *mageck test -h*.

For the RL2-based screen described in this protocol, the RL2-high population represents the phenotype-enriched population, whereas the corresponding unsorted sample is used as the reference group.

MAGeCK first evaluates the relative enrichment or depletion of individual sgRNAs between the selected and reference samples. RRA then evaluates whether multiple sgRNAs targeting the same gene are consistently enriched toward the same end of the ranked sgRNA list and generates a gene-level RRA score. A smaller RRA score indicates stronger statistical evidence in the corresponding selection direction.

The principal output files from the *mageck test* include:

*<output_prefix>.sgrna_summary.txt<output_prefix>.gene_summary.txt*

The *sgrna_summary.txt* file contains sgRNA-level ranking results, including normalized read counts, log2 fold change, P values, and FDR. The *gene_summary.txt* file contains gene-level ranking results, including the RRA score, P value, FDR, rank, number of supporting sgRNAs, and gene-level log2 fold-change information.

**4. Gene ranking and functional enrichment analysis**

Rank candidate genes according to the gene-level MAGeCK results. Consistent with the original RL2 screen, candidate genes were ranked primarily by RRA score, with FDR and log2 fold change used as supplementary criteria for filtering and interpretation [2]. The resulting gene-level ranking is shown in Figure 2C. Genes enriched in the RL2-high population represent candidate factors whose loss is associated with increased intracellular RL2 fluorescence.

From the genome-wide screen, 1,038 genes with nominal P < 0.05 were selected as the input for KEGG pathway enrichment analysis [2]. The threshold of nominal P < 0.05 was used as a preliminary filtering step to select genes for KEGG enrichment analysis, rather than as the final criterion for defining hit genes. The resulting KEGG pathway enrichment analysis is shown in Figure 2D. Enriched pathways included ECM–receptor interaction, thermogenesis, histidine metabolism, proteoglycans in cancer, and maturity-onset diabetes of the young.

## General notes and troubleshooting

**Troubleshooting**

**1. Low transformation efficiency during GeCKO library amplification**

Possible causes: Electrocompetent cells have lost efficiency during storage, agar plates are aged, or electroporation conditions are suboptimal.

Solutions: Use freshly thawed high-efficiency electrocompetent cells and freshly prepared ampicillin LB agar plates whenever possible. Confirm that the electroporation time constant is >3.5 ms. If the time constant is consistently low, repeat the electroporation using fresh cuvettes and increase the voltage to 1,800 V if the competent cells tolerate this setting.

**2. Low lentiviral titer**

Possible causes: Poor HEK293T cell health, suboptimal transfection efficiency, or low plasmid purity.

Solutions: Ensure that HEK293T cells are 70%–80% confluent at the time of transfection and free of mycoplasma contamination. Use highly pure endotoxin-free plasmid DNA for the GeCKO library and packaging plasmids. Fresh Lipofectamine reagent and Opti-MEM should be used for each transfection.

**3. Poor transduction efficiency during MOI determination or large-scale screening**

Possible causes: Polybrene degradation, inaccurate viral titer estimation, or excessive freeze/thaw cycles of lentivirus.

Solutions: Use freshly prepared Polybrene stock solution and verify a final concentration of 8 μg/mL. Avoid repeated freeze-thaw cycles of concentrated lentivirus. Re-titrate the virus if transduction efficiency deviates from the expected MOI 0.3–0.5 range.

**4. Excessive cell clumping during intracellular staining**

Possible causes: Cation-dependent cell adhesion, DNA release from dead cells, or antibody aggregates.

Solutions: Always use Ca^2+^/Mg^2+^-free DPBS during washing steps. Supplement the sorting buffer with 1 mM EDTA to disrupt cation-dependent aggregation. If clumping remains severe, add DNase I (25–50 μg/mL) together with 5 mM MgCl_2_ during the wash steps. Filtering the primary antibody solution through a 0.22 μm filter before staining is strongly recommended.

**5. High cell loss during fixation and permeabilization**

Possible causes: Excessive centrifugation force, harsh pipetting, or prolonged fixation.

Solutions: Reduce centrifugation speed to 400–500× *g* whenever possible and minimize pipetting steps. Resuspend pellets by gentle tube flicking rather than repeated pipetting. Do not fix cells in 4% PFA for longer than 15–20 min, as over-fixation increases fragility and reduces RL2 accessibility.

**6. Weak or absent RL2 intracellular signal**

Possible causes: Epitope masking due to over-fixation or suboptimal antibody dilution.

Solutions: Reduce fixation time to 10–15 min. Re-titrate the RL2 antibody within the 1:200-1:500 range, and always include a positive control sample known to have elevated *O*-GlcNAc levels.

**7. High background fluorescence during flow cytometry**

Possible causes: Nonspecific antibody binding or insufficient blocking.

Solutions: Increase BSA concentration in blocking and staining buffers to 2%–4%, extend the blocking step to 1 h, and use highly cross-adsorbed secondary antibodies. Additional wash steps may further reduce the background.

**8. PCR amplification failure or weak 170 bp sgRNA band**

Possible causes: Low genomic DNA input, incomplete reversal of PFA crosslinks, or degraded DNA.

Solution: Use 1–3 μg of genomic DNA per PCR reaction and distribute DNA across multiple parallel reactions. For fixed sorted cells, ensure that proteinase K digestion is performed for at least 12 h. Verify genomic DNA integrity before PCR whenever possible.

**9. Unexpected MAGeCK gene ranking or poor replicate concordance**

Possible causes: Biological variability, insufficient sequencing depth, inconsistent sorting gates, or stochastic sgRNA dropout.

Solutions: Use the two independent screens described in the original experiment and apply the same gating hierarchy and top-5% threshold in both screens. Candidate genes should only be prioritized when supported by multiple concordant sgRNAs across replicates.

## Validation of protocol

This protocol was used and validated in Zhang et al. [2], which identified FBXO31-mediated regulation of OGT and *O-*GlcNAcylation homeostasis in endometrial malignancy.
